# The Impact of Metabolic Syndrome on the Incidence of Atrial Fibrillation: A Nationwide Longitudinal Cohort Study in South Korea

**DOI:** 10.3390/jcm8081095

**Published:** 2019-07-24

**Authors:** Chang Hee Kwon, Hyeongsu Kim, Sung Hea Kim, Bum Sung Kim, Hyun-Joong Kim, Ji Dong Sung, Duk-Kyung Kim, Seong Woo Han, Kyu-Hyung Ryu

**Affiliations:** 1 Division of Cardiology, Department of Internal Medicine, Konkuk University Medical Center, Konkuk University School of Medicine, Seoul 05030, Korea; 2 Department of Preventive Medicine, School of Medicine, Konkuk University, Seoul 05030, Korea; 3 Division of Cardiology, Department of Medicine, Heart Vascular Stroke Institute, Samsung Medical Center, Sungkyunkwan University School of Medicine, Seoul 06351, Korea; 4 Division of Cardiology, Dongtan Sacred Heart Hospital, Hallym University College of Medicine, Hwaseong 18450, Korea

**Keywords:** atrial fibrillation, Korean, metabolic syndrome, risk factors

## Abstract

**Aims:** To evaluate the impact of metabolic syndrome (MetS) status on the incidence of atrial fibrillation (AF) in Koreans. **Methods and results:** Data obtained from the Korean National Health Insurance Service from 2009 to 2016 were analyzed. In total, 7,830,602 men and women (between 30 and 69 years of age) without baseline AF who underwent a national health examination between January 2009 and December 2009 were enrolled. Patients were evaluated to determine the impact of MetS status on their risk of developing AF until December 2016. Using the National Cholesterol Education Program Adult Treatment Panel III criteria, patients were placed into one of three groups depending on MetS component numbers: 0 (normal), 1–2 (Pre-MetS) or 3–5 (MetS). During a mean follow-up of 7.3 years, 20,708 subjects (0.26%) were diagnosed with AF. After multivariable adjustment, the risk of AF was significantly and positively correlated with MetS status (hazard ratios (HR) 1.391, 95% confidence interval (CI) 1.322–1.464 in Pre-MetS and HR 1.722, 95% CI 1.621–1.829 in MetS). When subgroup analyses were conducted according to MetS components, abdominal obesity (HR 1.316, *p* < 0.001), elevated blood pressure (HR 1.451, *p* < 0.001), and elevated fasting glucose (HR 1.163, *p* < 0.001) were associated with an increased risk of AF. **Conclusion:** MetS and pre-MetS are significantly associated with an increased risk of AF in Korean adults. Of the MetS components, abdominal obesity, elevated blood pressure, and elevated fasting glucose are potent risk factors for the risk of AF in this population.

## 1. Introduction

Atrial fibrillation (AF) is the most common cardiac arrhythmia and is independently associated with increased risks of mortality and morbidity [1,2,3]. Thus, identification of risk factors is important to help prevent AF and guide therapeutic decisions. Many clinical risk factors have been reported for the development of AF (e.g., increasing age, hypertension (HTN), diabetes mellitus, myocardial infarction, valvular heart disease, heart failure, obesity, obstructive sleep apnea, alcohol use, hyperthyroidism, and stress) [4,5,6,7,8,9].

Metabolic syndrome (MetS) is a cluster of metabolic risk factors including abdominal obesity, hypertension (HTN), hyperglycemia, and dyslipidemia; several definitions have been suggested using different criteria [10,11,12]. Previous studies have reported that MetS is associated with an increased risk of AF [13,14,15,16], however, these studies were conducted using: (i) western populations [14,15], (ii) body mass index (BMI) in place of waist circumference (WC) for abdominal obesity [13], and (iii) populations of only middle-aged men [16].

In this nationwide longitudinal cohort study, we used the Korean National Health Insurance Service (NHIS) database to assess the association between MetS status and incidence of AF among the general population.

## 2. Methods

### 2.1. Study Population

The NHIS provides mandatory health insurance for all South Korean citizens, covering nearly all forms of health care (e.g., health screening examinations for employed and self-employed insured individuals aged 20 years or older, all dependents aged 40 years or older) [17]. The NHIS database includes data on demographic characteristics, hospital admissions, outpatient department visits, pharmaceutical visits, and health screening examinations. Health screening examinations include information about health behavior obtained from a questionnaire, physical examinations, and blood tests. The Korean Industrial Safety and Health Law requires working individuals to participate in an annual or biennial health examination. All diagnoses are recorded in the NHIS database using the International Classification of Diseases-Tenth revision (ICD-10) codes.

**Figure 1:**
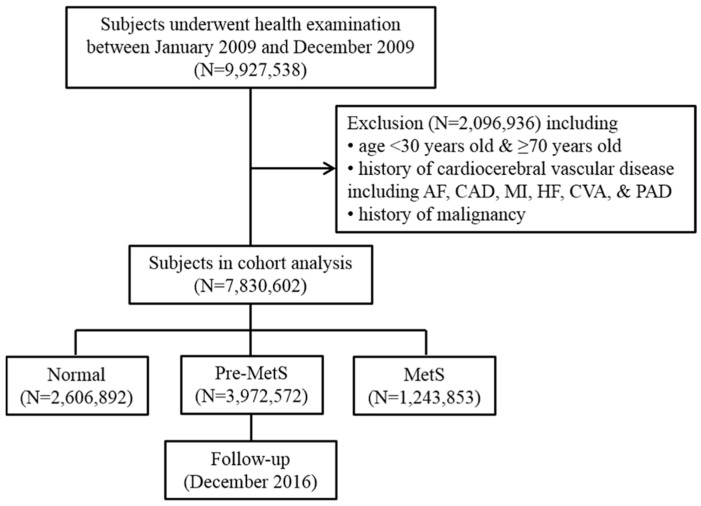
Flow diagram of the study population. AF indicates atrial fibrillation; CAD, coronary artery disease; CVA, cerebrovascular accident; HF, heart failure; MetS, metabolic syndrome; MI, myocardial infarction; and PAD, peripheral artery disease.

The study population assessed here included individuals who underwent health screening examinations between January 12,009 and December 12,009 in South Korea. Among the 9,927,538 participants, 2,096,936 were excluded for varying reasons (i.e., <30 years old or ≥70 years old, history of cardiocerebral vascular disease, history of malignancy (ICD-10 codes C00.X-C99.X)). The specific cardiocerebral vascular diseases or variables included AF (ICD-10 codes I48), coronary artery disease (procedure codes M6551-4), myocardial infarction (ICD-10 codes I21), heart failure (ICD-10 codes I42 or I50), cerebrovascular accident (ICD-10 codes I60.X-I609.X), and peripheral arterial disease (ICD-10 codes I73 or I74), which were diagnosed at tertiary hospital except AF. AF was diagnosed when the diagnostic code of AF occurred form any hospitals. The final study population consisted of 7,830,602 men and women. These subjects were then divided into three groups according to their number of MetS components: 0 (Normal group), 1–2 (pre-MetS group), and 3–5 MetS group (3–5). We analyzed follow-up data until December 31, 2016. Figure 1 presents a flow diagram of the study population. This study was approved by the Institutional Review Board of Konkuk University Medical Center. The requirement for informed consent was waived because data in the NHIS database are anonymized in adherence with strict confidentiality guidelines.

### 2.2. Definition of Metabolic Syndrome

MetS was defined according to the modified criteria of the National Cholesterol Education Program (NCEP) Adult Treatment Panel III (ATP III) criteria—the most commonly agreed-upon criteria [18]. A diagnosis of MetS was made when at least three of five components are present: (1) abdominal obesity (WC ≥ 90 cm for men, ≥ 85 cm for women); (2) elevated BP (systolic BP ≥ 130 mmHg or diastolic BP ≥ 85 mmHg or treatment of previously diagnosed HTN); (3) elevated fasting glucose (≥100 mg/dL or treatment of previously diagnosed DM); (4) high triglyceride (TG) (≥150 mg/dL or drug treatment for high TG); and (5) low high-density lipoprotein cholesterol (HDL-C) (<40 mg/dL for men, <50 mg/dL for women or drug treatment for low HDL-C). Subjects with one or two MetS components were defined as pre-MetS, and those with no MetS components were defined as normal.

### 2.3. Definition of Primary Outcome

The primary outcome of this analysis was the incidence of AF during follow-up. We defined an AF event using newly occurrence of the ICD-10 codes for AF (I48) and the prescription of an antiplatelet or anticoagulant regardless of admission or outpatient department visit.

### 2.4. Statistical Analyses

Follow up was initiated at the date of health screening examination and ended at the incidence of AF, or 31 December 2016, whichever came first. The following were considered covariates: age (categorical variable; 10 years), sex, smoking status (categorical variable; non-, ex-, or current smoker), alcohol consumption (categorical variable; no drink, 2–3 per month, 1–4 per week, or ≥5 per week), exercise (categorical variable; no, 1–4, or ≥5 per week), family history of HTN, DM, stroke, or heart disease, BMI (continuous variable), hemoglobin (continuous variable), creatinine (continuous variable), total cholesterol (continuous variable), low-density lipoprotein cholesterol (continuous variable), and alanine aminotransferase (continuous variable).

Baseline variables according to MetS status were compared using a chi-square test. Incidence rates (per 100,000 person-years) of AF by sex, age group, and MetS status were compared using a chi-square test.

Cox proportional hazard models were used to estimate hazard ratios (HR) and 95% confidence intervals (CI) for the incidence of AF during follow-up by MetS status. The censoring date was the earliest of the following: date of death, date of the primary outcome, or end date of the study period (31 December 2016). The models were initially unadjusted. Further adjustments were made for sex, age, smoking status, and exercise status (Model 1). Model 2 was adjusted as Model 1, and also for family history of HTN, stroke, heart disease, and DM. Model 3 was adjusted as Model 2 and also for BMI, hemoglobin, creatinine, total cholesterol, low-density lipoprotein cholesterol, and alanine aminotransferase.

Statistical significance was defined as a 2-sided *p*-value < 0.05. All analyses were conducted using SAS software (version 9.1; SAS Institute Inc., Cary, NC, USA).

## 3. Results

### 3.1. Baseline Characteristics

A total of 7,830,602 subjects were included in the cohort analysis. At baseline, prevalence of MetS was 1251, 138 (15.9%), and pre-MetS was present in 3,972,572 subjects (50.7%). Table 1 reveals baseline characteristics by MetS status. The prevalence of MetS significantly increased by increasing age groups. In those patients between 60 and 69, the prevalence of MetS was 25.2%. Males had an increased prevalence of pre-MetS and Mets compared with females. Additionally, the prevalence of MetS was significantly associated with the following variables: ex- or current smoker; higher alcohol intake; and family history of HTN, DM, stroke, or heart disease.

### 3.2. Association between MetS Status and AF Risk

**Figure 2:**
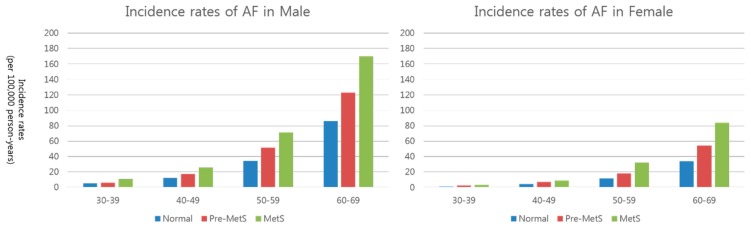
Association between metabolic syndrome status and risk of atrial figure according to patient sex and age. Subjects with pre-MetS or MetS have an increased risk of new-onset AF in all groups.

During the total follow-up period of 57,000,000 person-years, AF occurred in 20,708 subjects (0.26%). The incidence of AF according to MetS status was as follows: 3025 (0.12%) in the normal group, 11,070 (0.28%) in the pre-MetS group, and 6613 (0.53%) in the MetS group. Table 2 presents the incidence rates (per 100,000 person-years) of AF according to sex, age group, and MetS status. Although overall AF incidence rates were 36.41 per 100,000 person-years), AF incidence rates significantly increased according to MetS status in both sexes and in all age groups (Figure 2).

### 3.3. Impact of MetS Status and Components on the Risk of AF

Multivariable Cox regression analysis was performed to evaluate the association between MetS status and risk of AF (Table 3). The non-adjusted HRs for AF in subjects with pre-MetS and MetS were 2.43 (95% CI 2.313–2.554), and 4.616 (4.379–4.866), respectively. After multivariable adjustment (model 3), the risk of AF was significantly higher in subjects with pre-MetS (HR 1.391, 95% CI 1.322–1.464) and MetS (HR 1.722, 95% CI 1.621–1.829). A multivariable analysis revealed that males, older age groups, and those with a family history of HTN, stroke, or heart disease were significant predictors for the incidence of AF.

Among the components of MetS, abdominal obesity (HR 1.316, 95% CI 1.256–1.379), elevated BP (HR 1.451, 95% CI 1.4–1.505), and elevated fasting glucose (HR 1.163, 95% CI 1.123–1.205) were associated with an increased risk of AF in multivariable adjusted analysis including BMI (Table 4).

## 4. Discussion

The present study demonstrated that pre-MetS and MetS status significantly impacted the risk of AF among the general Korean population. During a mean follow-up of 7.3 years, subjects with pre-MetS had a 39.1% increased risk, while those with MetS had a 72.2% increased risk of new-onset AF. Among the components of MetS, abdominal obesity, elevated BP, and elevated fasting glucose were significant predictors for the development of new-onset AF.

### 4.1. Comparison to Previous Studies

In this study, the prevalence of MetS was 15.9%, lower than previous reports (31.3%) among participants in the Korean National Health and Nutrition Examination Surveys for 2007 [19]. This difference could be explained when considering that this study excluded subjects with a history of cardiocerebral vascular disease or malignancy and those ≥70 years old.

Previous studies have revealed a significant association between MetS and risk of AF, namely that subjects with MetS have an HR of 1.15–2.03 compared to those without MetS [13,14,15,16,20]. Of these studies, two were performed in Asian populations. A Japanese prospective, community-based, observational cohort study (The Niigata Preventive Medicine Study) reported that MetS, defined as the criteria of NCEP-ATP III, was associated with an increased risk of AF (age and sex-adjusted HR 1.88) [13]. The second study, in middle-aged Korean men, also revealed an association of MetS-defined using the International Diabetes Federation-had an increased risk of AF (multivariable-adjusted HR 1.57) [16]. Although all studies suggest that MetS is a risk factor for AF, previous studies adjusted for a limited number of variables; the study presented here considered more variables and included an adjusted analysis for a nationwide population. Thus, our results may serve to better describe the association between MetS and risk of AF compared to previous studies.

In the present study, abdominal obesity, elevated BP, and elevated fasting glucose were significantly associated with the incidence of AF, however, the influence of dyslipidemia components were low. In particular, abdominal obesity was associated with an increased risk (multivariable HR 1.316) of AF despite adjusting for BMI. Our results are consistent with those of previous studies using NHIS revealing that prehypertension, impaired fasting glucose, and abdominal obesity are important risk factors of AF [21,22].

### 4.2. MetS and AF

MetS and individual MetS components have been known to be risk factors for the incidence or recurrence of AF. Although the actual mechanisms of the relationship between MetS and AF remain unclear, several mechanisms have been proposed to explain the association between MetS and AF. Obesity, a primary component of MetS, was directly correlated with left atrial size [23,24] and increased left atrial size is an important precursor of left atrial remodeling, which is a critical component of AF development [25,26,27,28]. In addition, oxidative stress [29], chronic inflammation [29,30,31], neurohormonal activation [32], and obstructive sleep apnea may contribute to the development of AF [6].

Insulin resistance has been suggested as the fundamental pathophysiology responsible for MetS [29]. Insulin resistance may contribute to atrial electro-structural remodeling in the following ways: (i) worsening diastolic function and increasing atrial size [33]; (ii) endothelial dysfunction [34]; (iii) activation of many proinflammatory transcription factors and the generation of reactive oxygen species [35,36]; (iv) overactivation of the sympathetic nervous system [37]; and (v) upregulation of angiotensin II receptor expression [38].

### 4.3. Clinical Implications

Our study supports the notion that MetS and pre-MetS are significantly associated with new-onset AF in relatively healthy populations without cardiocerebral vascular disease. Two-thirds of the subjects in this cohort were defined as having MetS or pre-MetS and thus are considered at risk for experiencing AF. Among MetS components, abdominal obesity, elevated BP, and elevated fasting glucose have been revealed to significantly influence the incidence of AF. Importantly, these MetS components are potentially modifiable risk factors because previous studies have reported that obesity, high BMI or abdominal circumference, cause a host of metabolic and cardiovascular diseases (e.g., AF and diabetes) [39,40]. Therefore, for those with MetS and pre-MetS, tight control of abdominal obesity, blood pressure, and fasting glucose may be the crucial first step in preventing AF [21]. As such, aggressive management of risk factors and long-term sustained weight loss are associated with significant reductions of AF burden or recurrence [41,42]. Regular exercise, dietary salt restriction, consumption of high-protein and low glycemic index meals, calorie-controlled foods, and cessation of smoking and alcohol are mandatory for AF prophylaxis in subjects with MetS and pre-MetS. Moreover, randomized controlled trials for the effectiveness of MetS components control and AF incidence are warranted in the future.

### 4.4. Limitations

The present study had several limitations. First, it was a retrospective cohort study and thus associated with the inherent limitations of this type of analysis. Second, our study excluded subjects with cardiocerebral vascular disease, and we revealed an association between MetS status and AF risk in relatively healthy populations. Thus, we cannot generalize our results to high-risk populations. Third, we were unable to adjust for echocardiographic data which was not included in national health check-up. Left atrial size is strongly associated with risk of AF, and the effect of MetS might be eliminated once the left atrial sized is taken into account. Finally, the AF diagnosis was based on the newly developed AF code (I48) defined by the ICD-10 and the prescription of antiplatelet or anticoagulant during follow-up. Patients with the AF code, but without prescription of antiplatelet or anticoagulant, AF patients without AF code, and some asymptomatic AF patients, especially paroxysmal AF, may have been undetected in this analysis. Therefore, the diagnosis of new-onset AF may have been underestimated. However, our study had strengths in that it was a nationwide study with a large sample size and long-term follow-up periods. Thus, our results may be an important representation of the association of MetS and risk of AF among the general Korean population.

## 5. Conclusions

This nationwide longitudinal cohort demonstrated that pre-MetS and MetS were significantly associated with an increased risk of AF among the general Korean population. Of the MetS components, abdominal obesity, elevated BP, and elevated fasting glucose were significant predictors for the development of AF.

## Figures and Tables

**Table 1:** Baseline characteristics of study population according to metabolic syndrome status.

Variable	Normal *N* = 2,606,892 (33.3%)	Pre-MetS *N* = 3,972,572 (50.7%)	MetS *N* = 1,251,138 (15.9%)	*p*-Value
Age (years) at baseline				<0.001
30–39	796,857 (52.7)	624,638 (41.3)	91,002 (6.0)	
40–49	769,361 (35.5)	1,090,078 (50.4)	304,487 (14.1)	
50–59	705,192 (29.3)	1,285,531 (53.4)	418,382 (17.4)	
60–69	335,482 (19.3)	970,325 (55.7)	437,267 (25.2)	
Sex				<0.001
Male	1,139,011 (25.6)	2,415,161 (54.3)	892,010 (20.1)	
Female	1,467,881 (43.4)	1,557,411 (46.0)	359,128 (10.6)	
Smoking status				<0.001
Non-smoker	1,727,253 (39.1)	2,134,365 (48.3)	553,548 (12.5)	
Ex-smoker	264,442 (24.7)	581,953 (54.2)	226,601 (21.1.)	
Current smoker	597,385 (26.0)	1,233,704 (53.8)	463,565 (20.2.)	
Alcohol consumption				<0.001
No drink	1,260,556 (36.0)	1,745,471 (49.8)	497,220 (14.2.)	
2–3 per month	1,080,613 (33.9)	1,611,254 (50.5)	497,186 (15.6.)	
1–4 per week	538,841 (34.1)	795,190 (50.2)	248,610 (15.7)	
≥5 per week	45,971 (18.7)	135,990 (55.4)	63,366 (25.8)	
Exercise				
No exercise	1,216,659 (33.9)	1,803,595 (50.2)	569,220 (15.9)	
1–4 per week	538,841 (34.1)	795,190 (50.2)	248,610 (15.7)	
≥5 per week	828,890 (31.9)	1,342,260 (51.7)	423,684 (16.3)	
Family history of HTN				<0.001
Yes	231,065 (28.0)	42,583 (51.5)	169,292 (20.5)	
No	1,533,367 (34.1)	2,271,217 (50.5)	691,741 (15.4)	
Family history of DM				<0.001
Yes	206,381 (29.1)	358,590 (50.5)	144,595 (20.4)	
No	1,566,796 (33.8)	2,353,078 (50.7)	720,945 (15.5)	
Family history of stroke				<0.001
Yes	115,766 (27.4)	224,443 (53.1)	82,800 (19.6)	
No	1,656,733 (33.6)	2,486,810 (50.5)	792,319 (15.9)	
Family history of coronary heart disease				<0.001
Yes	80,356 (31.4)	131,259 (51.3)	44,348 (17.3)	
No	1,691,243 (33.2)	2,577,805 (50.7)	819,893 (16.1)	

Data are reported as number (%). DM, diabetes mellitus; HTN, hypertension; MetS, metabolic syndrome.

**Table 2:** Incidence rates (per 100,000 person-years) of atrial fibrillation according to sex, age group, and metabolic syndrome status.

Sex	MetS Status	Age Groups (Years)			
		30–39	40–49	50–59	60–69
Male	Normal	5.03	12.32	34.40	85.99
	Pre-MetS	6.20	17.31	51.48	122.95
	MetS	10.83	25.56	71.09	169.9
	*p*-value *	<0.001	<0.001	<0.001	<0.001
Female	No MetS	1.21	4.04	11.65	34.07
	Pre-MetS	2.18	7.19	18.24	54.22
	MetS	3.25	8.69	32.05	84.08
	*p-*value *	>0.05	<0.01	<0.001	<0.001

* *p*-Value among 3 groups. MetS, metabolic syndrome.

**Table 3:** Risk of atrial fibrillation: Cox proportional hazard model.

	Non-Adjusted HR (95% CI)	Multivariable HR * (95% CI)		
		Model 1	Model 2	Model 3
MetS status				
Normal	1	1	1	1
Pre-MetS	2.43 (2.313–2.554)	1.538 (1.463–1.617)	1.532 (1.457–1.611)	1.391 (1.322–1.464)
MetS	4.616 (4.379–4.866)	2.219 (2.103–2.343)	2.205 (2.089–2.328)	1.722 (1.621–1.829)
Sex				
Female		1	1	1
Male		2.332 (2.222–2.446)	2.358 (2.247–2.475)	1.969 (1.86–2.085)
Age group				
30–39		1	1	1
40–49		2.62 (2.327–2.95)	2.596 (2.305–2.923)	2.613 (2.319–2.944)
50–59		7.36 (6.58–8.233)	7.227 (6.461–8.085)	7.553 (6.748–8.455)
60–69		18.571 (16.622–20.747)	18.258 (16.341–20.4)	19.523 (17.46–21.83)
Smoking status				
Non-smoker		1	1	1
Ex-smoker		1.051 (1.0–1.105)	1.04 (0.99–1.094)	1.047 (0.996–1.101)
Current smoker		1.017 (0.971–1.065)	1.014 (0.969–1.062)	1.034 (0.987–1.083)
Exercise				
No exercise		1	1	1
1–4 per week		0.949 (0.906–0.994)	0.945 (0.902–0.99)	0.943 (0.9–0.987)
≥5 per week		1.009 (0.972–1.047)	1.003 (0.996–1.041)	0.99 (0.954–1.028)
Family history of HTN				
No			1	1
Yes			1.14 (1.09–1.193)	1.129 (1.079–1.181)
Family history of stroke				
No			1	1
Yes			1.139 (1.081–1.201)	1.145 (1.086–1.208)
Family history of coronary heart disease				
No			1	1
Yes			1.259 (1.173–1.352)	1.262 (1.175–1.356)
Family history of DM				
No			1	1
Yes			0.889 (0.844–0.937)	0.887 (0.841–0.935)
Body mass index (kg/m^2^)				1.069 (1.062–1.075)
Hemoglobin (g/dL)				1.082 (1.066–1.099)
Creatinine (mg/dL)				1.002 (0.992–1.012)
Total cholesterol (mg/dL)				0.997 (0.997–0.998)
LDL cholesterol (mg/dL)				1.0 (0.999–1.001)
ALT (IU/L)				0.999 (0.998–1.0)

* Multivariable adjusted HR = adjusted for sex, age, smoking status, exercise, family history of hypertension, stroke, diabetes mellitus, and heart disease, body mass index, hemoglobin, creatinine, total cholesterol, low-density lipoprotein cholesterol, and alanine aminotransferase, and other metabolic components. ALT, alanine aminotransferase; CI, confidence intervals; DM, diabetes mellitus; HR, hazard ratio; HTN, hypertension; LDL, low-density lipoprotein; MetS, metabolic syndrome.

**Table 4:** Risk of atrial fibrillation according to individual components of metabolic syndrome.

	Multivariable HR * (95% CI)	*p*-Value
Abdominal obesity	1.316 (1.256–1.379)	<0.0001
Elevated blood pressure	1.451 (1.4–1.505)	<0.0001
Elevated fasting glucose	1.163 (1.123–1.205)	<0.0001
High triglyceride	0.944 (0.907–0.984)	0.006
Low HDL cholesterol	1.048 (1.003–1.096)	0.038

* Multivariable adjusted HR = adjusted for sex, age, smoking status, exercise, family history of hypertension, stroke, diabetes mellitus, and heart disease, body mass index, hemoglobin, creatinine, total cholesterol, low-density lipoprotein cholesterol, and alanine aminotransferase, and other metabolic components. CI, confidence intervals; HDL, high-density lipoprotein; HR, hazard ratio.

## References

[1] Benjamin E.J., Wolf P.A., D’Agostino R.B., Silbershatz H., Kannel W.B., Levy D. (1998). Impact of Atrial Fibrillation on the Risk of Death: The Framingham Heart Study. Circulation.

[2] Stewart S., Hart C.L., Hole D.J., McMurray J.J. (2002). A Population-based Study of the Long-Term Risks Associated with Atrial Fibrillation: 20-Year Follow-up of the Renfrew/Paisley Study. Am. J. Med..

[3] Krahn A.D., Manfreda J., Tate R.B., Mathewson F.A., Cuddy T.E. (1995). The Natural History of Atrial Fibrillation: Incidence, Risk Factors, and Prognosis in the Manitoba Follow-up Study. Am. J. Med..

[4] Benjamin E.J., Levy D., Vaziri S.M., D’Agostino R.B., Belanger A.J., Wolf P.A. (1994). Independent Risk Factors for Atrial Fibrillation in a Population-Based Cohort. The Framingham Heart Study. JAMA.

[5] Frost L., Hune L.J., Vestergaard P. (2005). Overweight and obesity as risk factors for atrial fibrillation or Flutter: The danish diet, cancer, and health study. Am. J. Med..

[6] Gami A.S., Hodge D.O., Herges R.M., Olson E.J., Nykodym J., Kara T., Somers V.K. (2007). Obstructive Sleep Apnea, Obesity, and the Risk of Incident Atrial Fibrillation. J. Am. Coll. Cardiol..

[7] Kodama S., Saito K., Tanaka S., Horikawa C., Saito A., Heianza Y., Anasako Y., Nishigaki Y., Yachi Y., Iida K.T. (2011). Alcohol Consumption and Risk of Atrial Fibrillation: A Meta-Analysis. J. Am. Coll. Cardiol..

[8] Sawin C.T., Geller A., Wolf P.A., Belanger A.J., Baker E., Bacharach P., Wilson P.W., Benjamin E.J., D’Agostino R.B. (1994). Low Serum Thyrotropin Concentrations as a Risk Factor for Atrial Fibrillation in Older Persons. N. Engl. J. Med..

[9] Severino P., Mariani M.V., Maraone A., Piro A., Ceccacci A., Tarsitani L., Maestrini V., Mancone M., Lavalle C., Pasquini M. (2019). Triggers for Atrial Fibrillation: The Role of Anxiety. Cardiol. Res. Pract..

[10] Expert Panel on Detection, Evaluation, and Treatment of High Blood Cholesterol in Adults (2001). Executive summary of the third report of the national cholesterol education program (NCEP) expert panel on detection, evaluation, and treatment of high blood cholesterol in adults (Adult Treatment Panel III). JAMA.

[11] Grundy S.M., Cleeman J.I., Daniels S.R., Donato K.A., Eckel R.H., Franklin B.A., Gordon D.J., Krauss R.M., Savage P.J., Smith S.C. (2005). Diagnosis and Management of the Metabolic Syndrome: An American Heart Association/National Heart, Lung, and Blood Institute Scientific Statement. Circulation.

[12] Alberti K.G., Zimmet P., Shaw J., IDF Epidemiology Task Force Consensus Group (2005). The Metabolic Syndrome—A New Worldwide Definition. Lancet.

[13] Watanabe H., Tanabe N., Watanabe T., Darbar D., Roden D.M., Sasaki S., Aizawa Y. (2008). Metabolic Syndrome and Risk of development of Atrial Fibrillation: The Niigata Preventive Medicine Study. Circulation.

[14] Chamberlain A.M., Agarwal S.K., Ambrose M., Folsom A.R., Soliman E.Z., Alonso A. (2010). Metabolic Syndrome and Incidence of Atrial Fibrillation among Blacks and Whites in the Atherosclerosis Risk in Communities (ARIC) Study. Am. Heart J..

[15] Nystrom P.K., Carlsson A.C., Leander K., de Faire U., Hellenius M.L., Gigante B. (2015). Obesity, Metabolic Syndrome and Risk of Atrial Fibrillation: A Swedish, Prospective Cohort Study. PLoS ONE.

[16] Kim Y.G., Choi K.J., Han S., Hwang K.W., Kwon C.H., Park G.M., Won K.B., Ann S.H., Kim J., Kim S.J. (2018). Metabolic Syndrome and the Risk of New-Onset Atrial Fibrillation in Middle-Aged East Asian Men. Circ. J..

[17] Cheol Seong S., Kim Y.Y., Khang Y.H., Heon Park J., Kang H.J., Lee H., Do C.H., Song J.S., Hyon Bang J., Ha S. (2017). Data Resource Profile: The National Health Information Database of the National Health Insurance Service in South Korea. Int. J. Epidemiol..

[18] Alberti K.G., Eckel R.H., Grundy S.M., Zimmet P.Z., Cleeman J.I., Donato K.A., Fruchart J.C., James W.P., Loria C.M., Smith S.C. (2009). Harmonizing the Metabolic Syndrome: A Joint Interim Statement of the International Diabetes Federation Task Force on Epidemiology and Prevention; National Heart, Lung, and Blood Institute; American Heart Association; World Heart Federation; International Atherosclerosis Society; and International Association for the Study of Obesity. Circulation.

[19] Lim S., Shin H., Song J.H., Kwak S.H., Kang S.M., Won Yoon J., Choi S.H., Cho S.I., Park K.S., Lee H.K. (2011). Increasing Prevalence of Metabolic Syndrome in Korea: The Korean National Health and Nutrition Examination Survey for 1998–2007. Diabetes Care.

[20] Tanner R.M., Baber U., Carson A.P., Voeks J., Brown T.M., Soliman E.Z., Howard V.J., Muntner P. (2011). Association of the Metabolic Syndrome with Atrial Fibrillation among United States Adults (from the REasons for Geographic and Racial Differences in Stroke [REGARDS] Study). Am. J. Cardiol..

[21] Lee S.S., Ae Kong K., Kim D., Lim Y.M., Yang P.S., Yi J.E., Kim M., Kwon K., Bum Pyun W., Joung B. (2017). Clinical Implication of an Impaired Fasting Glucose and Prehypertension Related to New Onset Atrial Fibrillation in a Healthy Asian Population without Underlying Disease: A Nationwide Cohort Study in Korea. Eur. Heart. J..

[22] Baek Y.S., Yang P.S., Kim T.H., Uhm J.S., Park J., Pak H.N., Lee M.H., Joung B. (2017). Associations of Abdominal Obesity and New-Onset Atrial Fibrillation in the General Population. J. Am. Heart Assoc..

[23] Wang T.J., Parise H., Levy D., D’Agostino R.B., Wolf P.A., Vasan R.S., Benjamin E.J. (2004). Obesity and the Risk of New-Onset Atrial Fibrillation. JAMA.

[24] Ayer J.G., Almafragy H.S., Patel A.A., Hellyer R.L., Celermajer D.S. (2008). Body Mass Index Is an Independent Determinant of Left Atrial Size. Heart Lung Circ..

[25] Wang W., Zhang F., Xhen J., Chen X., Fu F., Tang M., Chen L. (2014). P-Wave Dispersion and Maximum Duration Are Independently Associated with Insulin Resistance in Metabolic Syndrome. Ann. Endocrinol. (Paris).

[26] Abed H.S., Samuel C.S., Lau D.H., Kelly D.J., Royce S.G., Alasady M., Mahajan R., Kuklik P., Zhang Y., Brooks A.G. (2013). Obesity Results in Progressive Atrial Structural and Electrical Remodeling: Implications for Atrial Fibrillation. Heart Rhythm.

[27] Munger T.M., Dong Y.X., Masaki M., Oh J.K., Mankad S.V., Borlaug B.A., Asirvatham S.J., Shen W.K., Lee H.C., Bielinski S.J. (2012). Electrophysiological and Hemodynamic Characteristics Associated with Obesity in Patients with Atrial Fibrillation. J. Am. Coll. Cardiol..

[28] Yasar A.S., Bilen E., Bilge M., Ipek G., Ipek E., Kirbas O. (2009). P-Wave Duration and Dispersion in Patients with Metabolic Syndrome. Pacing Clin. Electrophysiol..

[29] Dandona P., Aljada A., Chaudhuri A., Mohanty P., Garg R. (2005). Metabolic Syndrome: A Comprehensive Perspective Based on Interactions between Obesity, Diabetes, and Inflammation. Circulation.

[30] Visser M., Bouter L.M., McQuillan G.M., Wener M.H., Harris T.B. (1999). Elevated C-Reactive Protein Levels in Overweight and Obese Adults. JAMA.

[31] Guo Y., Lip G.Y., Apostolakis S. (2012). Inflammation in Atrial Fibrillation. J. Am. Coll. Cardiol..

[32] Sarzani R., Salvi F., Dessi-Fulgheri P., Rappelli A. (2008). Renin-Angiotensin System, Natriuretic Peptides, Obesity, Metabolic Syndrome, and Hypertension: An Integrated View in Humans. J. Hypertens..

[33] Dinh W., Lankisch M., Nickl W., Scheyer D., Scheffold T., Kramer F., Krahn T., Klein R.M., Barroso M.C., Futh R. (2010). Insulin Resistance and Glycemic Abnormalities are Associated with Deterioration of Left Ventricular Diastolic Function: A Cross-Sectional Study. Cardiovasc. Diabetol..

[34] Kravariti M., Naka K.K., Kalantaridou S.N., Kazakos N., Katsouras C.S., Makrigiannakis A., Paraskevaidis E.A., Chrousos G.P., Tsatsoulis A., Michalis L.K. (2005). Predictors of Endothelial Dysfunction in Young Women with Polycystic Ovary Syndrome. J. Clin. Endocrinol. Metab..

[35] Aljada A., Ghanim H., Mohanty P., Kapur N., Dandona P. (2002). Insulin Inhibits the Pro-Inflammatory Transcription Factor Early Growth Response Gene-1 (Egr)-1 Expression in Mononuclear Cells (MNC) and Reduces Plasma Tissue Factor (TF) and Plasminogen Activator Inhibitor-1 (PAI-1) Concentrations. J. Clin. Endocrinol. Metab..

[36] Dandona P., Aljada A., Mohanty P., Ghanim H., Hamouda W., Assian E., Ahmad S. (2001). Insulin Inhibits Intranuclear Nuclear Factor KappaB and Stimulates IkappaB in Mononuclear Cells in Obese Subjects: Evidence for an Anti-Inflammatory Effect?. J. Clin. Endocrinol. Metab..

[37] Grassi G., Dell’Oro R., Quarti-Trevano F., Scopelliti F., Seravalle G., Paleari F., Gamba P.L., Mancia G. (2005). Neuroadrenergic and Reflex Abnormalities in Patients with Metabolic Syndrome. Diabetologia.

[38] Yamagishi S.I., Matsui T., Nakamura K. (2008). Possible Molecular Mechanisms by Which Angiotensin II Type 1 Receptor Blockers (ARBs) Prevent the Development of Atrial Fibrillation in Insulin Resistant Patients. Horm. Metab. Res..

[39] Vaziri S.M., Larson M.G., Lauer M.S., Benjamin E.J., Levy D. (1995). Influence of Blood Pressure on Left Atrial Size. The Framingham Heart Study. Hypertension.

[40] Aronis K.N., Wang N., Phillips C.L., Benjamin E.J., Marcus G.M., Newman A.B., Rodondi N., Satterfield S., Harris T.B., Magnani J.W. (2015). Associations of Obesity and Body Fat Distribution with Incident Atrial Fibrillation in the Biracial Health Aging and Body Composition Cohort of Older Adults. Am. Heart J..

[41] Pathak R.K., Middeldorp M.E., Lau D.H., Mehta A.B., Mahajan R., Twomey D., Alasady M., Hanley L., Antic N.A., McEvoy R.D. (2014). Aggressive Risk Factor Reduction Study for Atrial Fibrillation and Implications for the Outcome of Ablation: The ARREST-AF Cohort Study. J. Am. Coll. Cardiol..

[42] Pathak R.K., Middeldorp M.E., Meredith M., Mehta A.B., Mahajan R., Wong C.X., Twomey D., Elliott A.D., Kalman J.M., Abhayaratna W.P. (2015). Long-Term Effect of Goal-Directed Weight Management in an Atrial Fibrillation Cohort: A Long-Term Follow-Up Study (LEGACY). J. Am. Coll. Cardiol..

